# DNA Damage Induces Dynamic Associations of BRD4/P-TEFb With Chromatin and Modulates Gene Transcription in a BRD4-Dependent and -Independent Manner

**DOI:** 10.3389/fmolb.2020.618088

**Published:** 2020-12-04

**Authors:** Yawei Song, Gongcheng Hu, Jinping Jia, Mingze Yao, Xiaoshan Wang, Wenliang Lu, Andrew P. Hutchins, Jiekai Chen, Keiko Ozato, Hongjie Yao

**Affiliations:** ^1^School of Life Sciences, University of Science and Technology of China, Hefei, China; ^2^CAS Key Laboratory of Regenerative Biology, Joint School of Life Sciences, Guangzhou Institutes of Biomedicine and Health, Chinese Academy of Sciences, Guangzhou Medical University, Guangzhou, China; ^3^Bioland Laboratory (Guangzhou Regenerative Medicine and Health GuangDong Laboratory), Guangzhou, China; ^4^Institute of Stem Cell and Regeneration, Chinese Academy of Sciences, Beijing, China; ^5^Guangdong Provincial Key Laboratory of Stem Cell and Regenerative Medicine, Guangzhou Institutes of Biomedicine and Health, Chinese Academy of Sciences, Guangzhou, China; ^6^Department of Biology, Southern University of Science and Technology, Shenzhen, China; ^7^Division of Developmental Biology, National Institute of Child Health and Human Development, Bethesda, MD, United States

**Keywords:** BRD4, P-TEFb, gene transcription, UV stress, JNK pathway

## Abstract

The bromodomain-containing protein BRD4 has been thought to transmit epigenetic information across cell divisions by binding to both mitotic chromosomes and interphase chromatin. UV-released BRD4 mediates the recruitment of active P-TEFb to the promoter, which enhances transcriptional elongation. However, the dynamic associations between BRD4 and P-TEFb and BRD4-mediated gene regulation after UV stress are largely unknown. In this study, we found that BRD4 dissociates from chromatin within 30 min after UV treatment and thereafter recruits chromatin. However, P-TEFb binds tightly to chromatin right after UV treatment, suggesting that no interactions occur between BRD4 and P-TEFb within 30 min after UV stress. *BRD4* knockdown changes the distribution of P-TEFb among nuclear soluble and chromatin and downregulates the elongation activity of RNA polymerase II. Inhibition of JNK kinase but not other MAP kinases impedes the interactions between BRD4 and P-TEFb. RNA-seq and ChIP assays indicate that BRD4 both positively and negatively regulates gene transcription in cells treated with UV stress. These results reveal previously unrecognized dynamics of BRD4 and P-TEFb after UV stress and regulation of gene transcription by BRD4 acting as either activator or repressor in a context-dependent manner.

## Introduction

During mitosis, most transcription factors are dissociated from chromosomes; however, BRD4 persistently associates with chromatin in interphase and chromosomes during mitosis to transmit epigenetic memory across cell divisions (Dey et al., 2003), indicating that BRD4 is involved in epigenetic memory. BRD4 binds to acetylated histones, resulting in dramatic decondensation of nearby chromatin. BRD4 stimulates G1 gene transcription by binding to multiple G1 gene promoters, allowing cells to progress toward S phase (Yang et al., 2008; Dey et al., 2009). The treatment of cells with anti-mitotic drugs, including nocodazole, results in BRD4 release from mitotic chromosomes (Nishiyama et al., 2012). Therefore, BRD4 plays important roles in maintaining epigenetic regulation and chromatin structure in cells. BRD4 also plays important roles in regulating gene expression through phase separation, and it forms nuclear puncta with MED1 at super-enhancers that exhibit properties of liquid-like condensates (Sabari et al., 2018).

BRD4 has been reported to be a drug target for several cancers (Qin et al., 2019; White et al., 2019). Indeed, inhibition of BRD4 by inhibitors revealed the involvement of BRD4 in various cancers in animal models (Zuber et al., 2011; Herrmann et al., 2012; Lockwood et al., 2012; Ott et al., 2012; Asangani et al., 2014; Segatto et al., 2017). However, the molecular mechanisms by which BRD4 mediates gene regulation remain under investigation. BRD4 recruits P-TEFb (composed of Cyclin T and CDK9) to stimulate RNA polymerase II (RNAPII) activity and support lineage-specific gene transcription (Jang et al., 2005; Yang et al., 2005; Zippo et al., 2009; Zhang et al., 2012). BRD4 has also been shown to regulate ERα-responsive gene expression by affecting the serine 2 phosphorylation of the C-terminal domain (CTD) of RNAPII and histone H2B monoubiquitination (Nagarajan et al., 2014).

As DNA damage alters chromatin structure and activates the specific signaling pathways that block cell-cycle progression (Jackson and Bartek, 2009; Misteli and Soutoglou, 2009), DNA damage can result in carcinogenesis (Shen et al., 2019). Through bromodomain interactions, BRD4 recruits the condensin II chromatin remodeling complex to acetylated histones, functioning as an endogenous inhibitor of the DNA damage response (Floyd et al., 2013). The DNA damage signal-triggered deacetylation of nucleosomal histone H4 at acetylated lysine 5 and 8 results in the release of chromatin-bound BRD4, which is essential for the recruitment of the active P-TEFb to the promoter to enhance transcription at the stage of elongation (Ai et al., 2011). However, the dynamic behaviors of BRD4 and P-TEFb and their associations with chromatin at the early stage after DNA damage, such as with UV irradiation, and the involved possible mechanisms are still unclear.

In this study, we investigated the dynamic associations of BRD4 and its associated protein complex P-TEFb with chromatin after UV stress. We observed that BRD4 dissociates from chromatin within 30 min after UV treatment and then recruits chromatin later on. However, P-TEFb binds tightly to chromatin immediately after UV treatment. *BRD4* knockdown results in the higher level of P-TEFb in both nuclear soluble fraction and chromatin fraction. *BRD4* knockdown reduces UV-stimulated elongating activity of RNA polymerase II. The JNK pathway is involved in the interactions between BRD4 and P-TEFb. Our data further indicated that BRD4 both positively and negatively regulates transcription of the specific genes in a context-dependent manner after UV stress.

## Results

### Determination of the Optimum Salt Concentration to Separate BRD4 Into Nuclear Soluble and Chromatin Fractions After UV Treatment

Salt (NaCl) and other reagents (ethidium bromide, spermine, and distamycin A) have been used to monitor the binding of proteins to chromatin (Lichota and Grasser, 2001). To obtain biochemical evidence for the interaction of BRD4/P-TEFb with chromatin *in vivo*, we performed differential salt extraction experiments using concentrations from 50 to 200 mM. After a preliminary screen, we chose NaCl at 100, 150, and 200 mM for further investigation (Supplementary Figure 1). Our results indicated that 100 mM NaCl was not sufficient to extract BRD4 into the nuclear soluble fraction, whereas 200 mM NaCl was too high to observe differences at different time points after UV treatment. However, 150 mM NaCl extracted different amounts of BRD4 and Cyclin T1 (a subunit of P-TEFb) into the nuclear soluble fraction after UV treatment at different time points. These results indicated that 150 mM NaCl was sufficient to explore the dynamic association of BRD4/P-TEFb with chromatin, therefore, we selected 150 mM NaCl for the ensuing experiments.

### Dynamic Associations of BRD4, P-TEFb, and RNAPII With Chromatin After UV Stress

**FIGURE 1:**
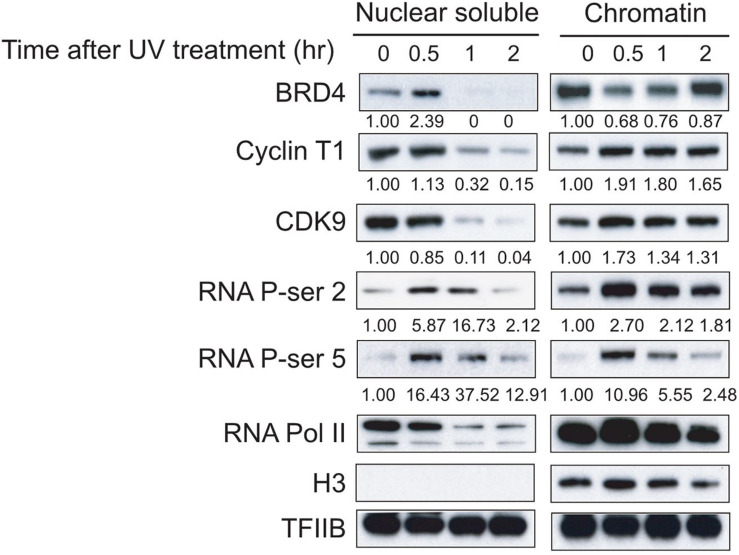
Dynamic associations of BRD4, P-TEFb, and RNAPII with chromatin after UV treatment. Nuclear soluble fraction and chromatin fraction were isolated from UV-treated cells at different time points (0, 0.5, 1, and 2 h). Nuclear soluble fraction and chromatin fraction were lysed in SDS-loading buffer and analyzed by Western blot to determine the amount of BRD4, Cyclin T1, and CDK9 remaining in nuclear fraction and chromatin fraction, and the dynamics of total RNAPII and Ser5 and Ser2 phosphorylation from both nuclear soluble fraction and chromatin fraction. An anti-histone H3 antibody was used as a loading control for chromatin fraction. An anti-TFIIB antibody was used as the loading control for both nuclear soluble fraction and chromatin fraction.

We took advantage of our established protein isolation condition and investigated the dynamic distributions of BRD4, P-TEFb, RNAPII, and phosphorylated CTD at serine (Ser) 2 and Ser 5 of RNAPII. We observed an increase in BRD4 in the nuclear soluble fraction and a decrease in BRD4 in the chromatin fraction at 30 min after UV treatment, indicating that UV stress disrupts the association between BRD4 and chromatin within 30 min (Figure 1). Interestingly, we found that a significant reduction of BRD4 in the nuclear soluble fraction corresponded to an increase in the chromatin fraction at 1 and 2 h after UV treatment (Figure 1). However, Cyclin T1 and CDK9 (two components of transcription elongation complex P-TEFb) levels gradually decreased in the nuclear soluble fraction and increased in the chromatin fraction after UV treatment. These results indicated that UV treatment significantly enhanced the association between P-TEFb and chromatin (Figure 1).

The CTD of the largest subunit of RNAPII plays a critical role in gene regulation and is composed of multiple heptad repeats (YSPTSPS motifs, 52 in mammals), each containing two main phospho-acceptor sites: Ser2 and Ser5. Phosphorylation of Ser5 occurs primarily at the 5′ region of genes, whereas Ser2 phosphorylation accumulates on the elongating polymerase and therefore is more abundant at the 3′ region of genes (Komarnitsky et al., 2000). We found a reduction in total RNAPII level in the nuclear soluble fraction in response to an elevation of total RNAPII level in the chromatin fraction within 30 min after UV treatment, which is similar to the dynamic distribution of P-TEFb (Figure 1). Ser5 and Ser2 phosphorylations of RNAPII CTD in both the nuclear soluble and chromatin fractions increased within 30 min after UV treatment and then gradually decreased, suggesting that UV damage shut down the general transcription of genes in cells incrementally from 30 min to 2 h after UV treatment (Figure 1).

### *BRD4* Knockdown Reduces the Association Between P-TEFb and Chromatin in Response to UV Stress

**FIGURE 2:**
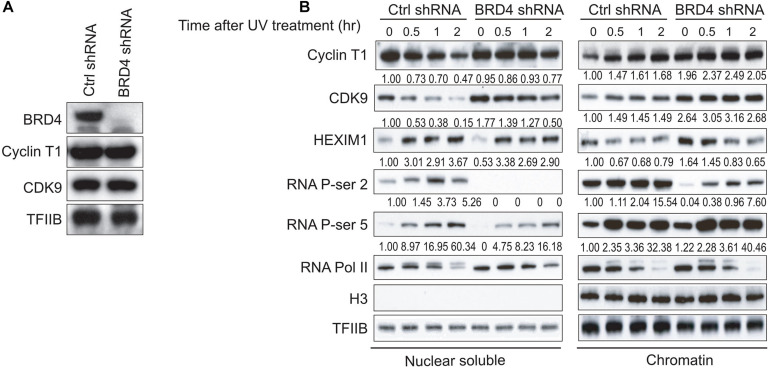
*BRD4* knockdown results in differential associations of P-TEFb, HEXIM1, and Ser2 phosphorylation of RNAPII CTD with chromatin. **(A)** Western blot analysis of BRD4 and P-TEFb levels in NEs from HeLa-S3 cells stably expressing *BRD4* shRNA or empty vector. TFIIB was used as a loading control. **(B)** Nuclear soluble fraction and chromatin fraction were isolated from control cells or BRD4-depleted cells treated with UV at different time points (0, 0.5, 1, and 2 h) and immunoblotted with antibodies (anti-CyclinT1, anti-CDK9, anti-HEXIM1, anti-RNAPII, anti-RNAPII phosphorylated at Ser5, anti-RNAPII phosphorylated at Ser2, anti-histone H3, and anti-TFIIB), as shown on the left. An anti-histone H3 antibody was used as a loading control for chromatin fraction. An anti-TFIIB antibody was used as the loading control for both nuclear soluble fraction and chromatin fraction.

BRD4 interacts with P-TEFb and increases the P-TEFb-dependent CTD phosphorylation at Ser2 of RNAPII and stimulates transcription from promoters *in vivo* (Jang et al., 2005; Yang et al., 2005). To further investigate whether BRD4 regulates the stability of P-TEFb with chromatin after UV stress, HeLa-S3 cells were first transduced with either control shRNA or *BRD4* shRNA lentiviral particles. Western blot indicated that the *BRD4* knockdown efficiency was very high in BRD4-depleted HeLa-S3 cells compared with the control shRNA-transduced cells (Figure 2A). Interestingly, Cyclin T1 did not show clear reduction in BRD4-depleted cells compared with control cells before UV treatment (Figure 2A), however, after UV treatment, we found that 150 mM NaCl extracted more Cyclin T1 and CDK9 proteins into the nuclear soluble fraction of the BRD4-depleted HeLa-S3 cells (lanes 7 and 8) than that of the control cells (lanes 3 and 4) (Figure 2B). We further discovered that the levels of both Cyclin T1 and CDK9 were much higher in the chromatin fraction of the BRD4-depleted cells than that of the control cells, suggesting that the binding of P-TEFb to soluble chromatin in cells was weaker after *BRD4* knockdown. P-TEFb (Cyclin T1 and CDK9) levels in the chromatin fraction in BRD4-depleted cells were higher than those in control shRNA-treated cells after UV treatment. However, compared with control shRNA-treated cells, P-TEFb levels in the chromatin fraction in the BRD4-depleted cells was not significantly increased from 30 min to 2 h after UV treatment (Figure 2B).

### *BRD4* Knockdown Increases HEXIM1 Association With Chromatin, but UV Stress Releases HEXIM1 From Chromatin

HEXIM1 was shown to inhibit P-TEFb in a 7SK-dependent manner and was released from P-TEFb when cells were treated with certain stresses, causing an increase in the level of active P-TEFb for stress-induced transcription (Yik et al., 2003). *BRD4* knockdown increased the level of HEXIM1 in the chromatin fraction (Figure 2B), suggesting that BRD4 and HEXIM1 are mutually exclusive with regard to chromatin binding. These data are consistent with the association of P-TEFb with either BRD4 or HEXIM1. In addition, the HEXIM1 protein levels gradually decreased in the chromatin fraction and gradually increased in the nuclear soluble fraction within 2 h after UV treatment (Figure 2B).

### *BRD4* Knockdown Significantly Reduces UV Stress-Activated CTD Phosphorylation at Ser2 of RNAPII

Next, we investigated whether BRD4 regulates UV stress-mediated RNAPII activity and CTD phosphorylation at Ser2 and Ser5. *BRD4* knockdown had no effect on the general activity of RNAPII but resulted in slight increases in the phosphorylation of CTD at Ser5 in the chromatin fraction, with a reduction in the nuclear soluble fraction. We found that *BRD4* knockdown notably reduced the UV-mediated phosphorylation of RNAPII CTD at Ser2, suggesting that BRD4 is required for the UV-mediated activation of phosphorylation of CTD at Ser2 (Figure 2B).

### The JNK Pathway Is Involved in the Interaction Between BRD4 and P-TEFb After UV Stress

**FIGURE 3:**
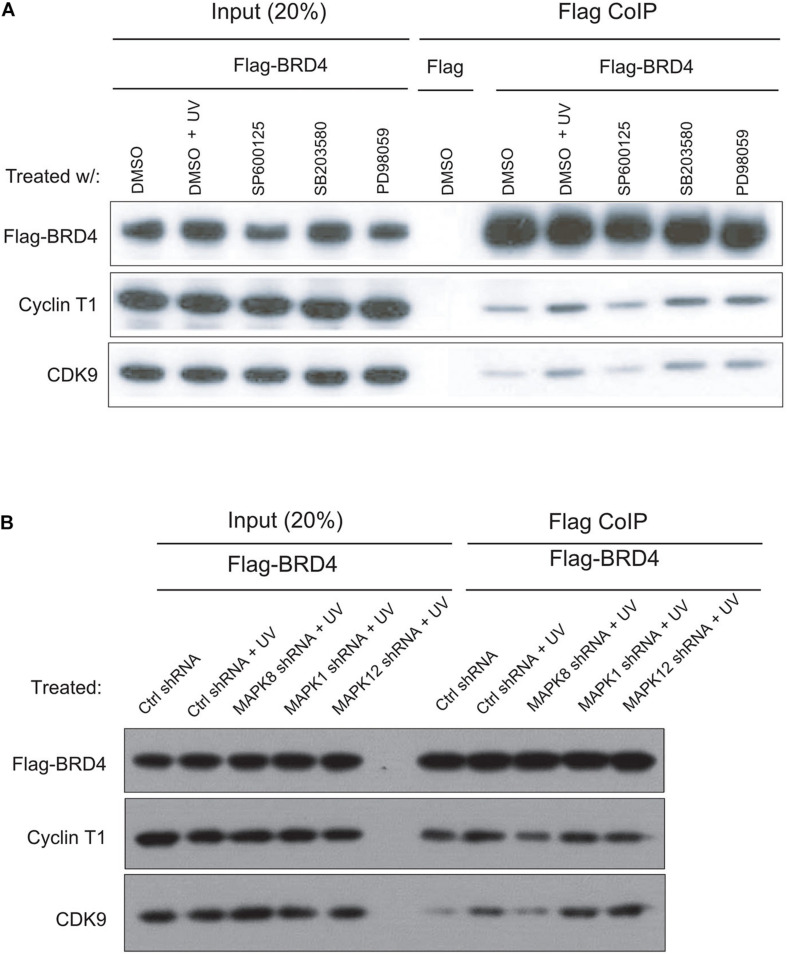
JNK inhibitor blocks UV-stimulated interactions between BRD4 and P-TEFb. **(A)** SP600125 (10 μM) or PD98059 or SB20458 (100 μM) was added to cells 2 h prior to UV treatment. The inhibitors were present during the subsequent 2 h of UV treatment. Then, HeLa-S3 NEs were extracted and precipitated with an anti-Flag M2 antibody, as shown on the top (IP), and the precipitates were immunoblotted with antibodies, as shown on the left. **(B)** shRNA-mediated knockdown of ERK, p38, and JNK in pOZN-Flag-*BRD4* stably expressed HeLa-S3 cells with treatment by UV. Then, HeLa NEs were extracted and precipitated with an anti-Flag M2 antibody, as shown on the top (IP), and the precipitates were immunoblotted with antibodies, as shown on the left.

It has been shown that anti-mitotic drug-induced BRD4 release depends on the activation of the JNK pathway, a MAPK downstream pathway (Nishiyama et al., 2012). To investigate whether a specific MAPK pathway is involved in interactions between BRD4 and P-TEFb after UV treatment, we examined several MAPK inhibitors. It has been reported that PD98059 is an inhibitor of MEK activity in the ERK pathways and that SB203580 is an inhibitor of p38 MAP kinase, SP600125 inhibits JNK activity (Barancík et al., 2001; Bennett et al., 2001; Gaillard et al., 2005). We added these three different inhibitors, respectively, to Flag-*BRD4* overexpressed HeLa-S3 cells 2 h prior to UV treatment. We then collected cells at 2 h after UV stress, prepared nuclear extracts, and performed Flag co-immunoprecipitation experiments. Based on the above data, we concluded that P-TEFb binds tightly to chromatin within 2 h after UV treatment and that BRD4 associates with chromatin at 2 h after UV treatment. Our co-immunoprecipitation experiments indicated that P-TEFb interacts more strongly with BRD4 after UV treatment in contrast to the control (Figure 3A). We found that pretreating cells with SB203580 and PD98059 before UV treatment results in similar binding patterns when compared with UV treatment. Pretreatment of cells with the JNK inhibitor SP600125 prior to UV stress significantly blocked the interactions between P-TEFb and BRD4 (Figure 3A). Meanwhile, we designed shRNA(s) targeting the major protein in one of the MAPK pathways (ERK, p38, and JNK) and established shRNA-mediated ERK, p38, and JNK-depleted stable cell lines, respectively, in pOZN-Flag-*BRD4* HeLa-S3 cell lines. Similarly, these data suggested that activation of JNK pathway by UV stress is required for the interaction of P-TEFb with BRD4 (Figure 3B).

### RNA-seq Analysis Reveals That UV-Responsive Genes Are Partially Regulated by BRD4

**FIGURE 4:**
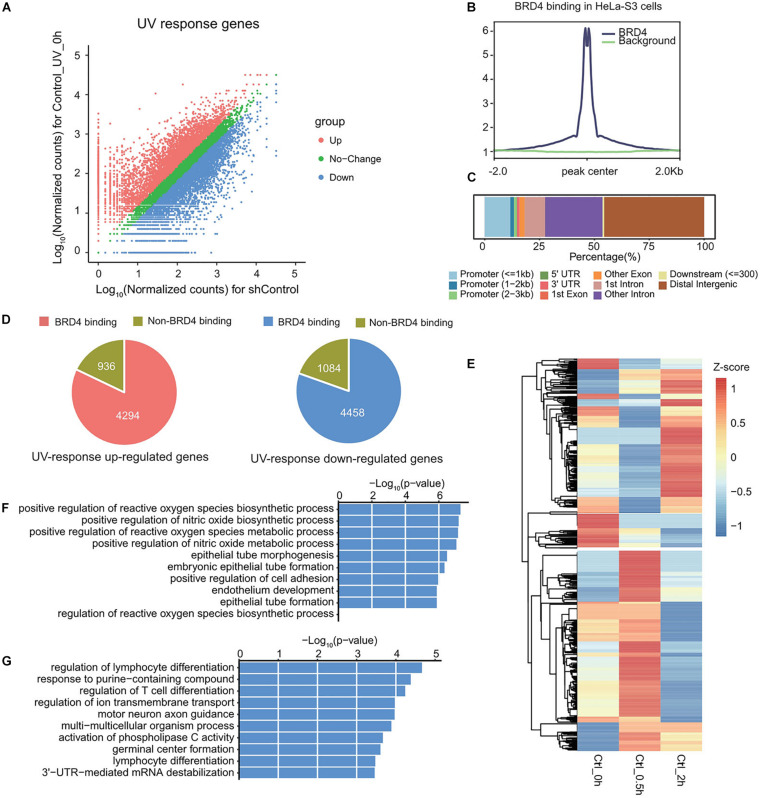
UV-responsive genes are partially regulated by BRD4. **(A)** Scatter plot showing the differentially expressed genes upon UV treatment in HeLa-S3 cells. **(B)** The average ChIP enrichment in BRD4 binding sites. **(C)** The genomic distribution of BRD4 binding sites in HeLa-S3 cells. Genome features are color-coded in the legend bar. The *x*-axis shows the cumulative percentage of genomic occupancy of each feature. **(D)** Pie charts showing the percentage of BRD4-regulated genes in UV-responsive genes. **(E)** Heatmap representing the expression pattern of UV-responsive/BRD4-regulated genes upon UV treatment. **(F)** Gene ontology analysis of genes in cluster 1 from **(D)**. **(G)** Gene ontology analysis of genes in cluster 2 from **(D)**.

To study the role of BRD4 in UV-regulated genome-wide gene expression, RNA samples from control and BRD4-depleted cells at 0, 0.5, and 2 h after UV treatment were analyzed using RNA-sequencing (RNA-seq). UV-responsive genes were identified by comparing RNA-seq data between UV-treated and untreated cells (GSE137850). Genes with fold change higher than 1.5-fold were regarded as significant and defined as UV-responsive genes (Figure 4A). To explore which genes might be targeted by BRD4, BRD4 chromatin immunoprecipitation (ChIP)-seq data in HeLa-S3 cells were analyzed (GSM1249906) (Liu et al., 2013). The normalized signal in BRD4 binding regions was significantly higher than that in background regions (Figure 4B). Then, BRD4 binding sites were annotated to gene regions, and results showed that more than 50% of BRD4 binding sites were located at gene promoters and gene bodies (Figure 4C), indicating that BRD4 binding at these sites might directly regulate gene expression. Therefore, the genes bound by BRD4 were chosen as BRD4-regulated genes. Overlapping between BRD4-regulated genes with UV-responsive genes showed that nearly 80% of UV-responsive genes have BRD4 binding (Figure 4D), and the overlapped genes were defined as UV-responsive/BRD4-regulated genes.

To further investigate BRD4-regulated UV-responsive genes, hierarchical clustering was performed (Figure 4E). In general, these genes could be divided into two categories, and each category accounted for nearly half of these genes. The genes in cluster 1 were upregulated at 0.5 h, and then downregulated at 2 h. GO analysis showed that biological function of these genes was mainly associated with metabolism (Figure 4F). In contrast, the genes in cluster 2 were downregulated at 0.5 h and upregulated at 2 h, and the expression pattern of these genes was consistent with dynamic chromatin-association of BRD4 after UV treatment, indicating that these genes might be directly regulated by BRD4. GO analysis showed that they were involved in the processes associated with cell development (Figure 4G).

### Dynamic Recruitments of BRD4, P-TEFb, and RNAPII to BRD4 Positively Regulated Gene *GATA3* After UV Stress

**FIGURE 5:**
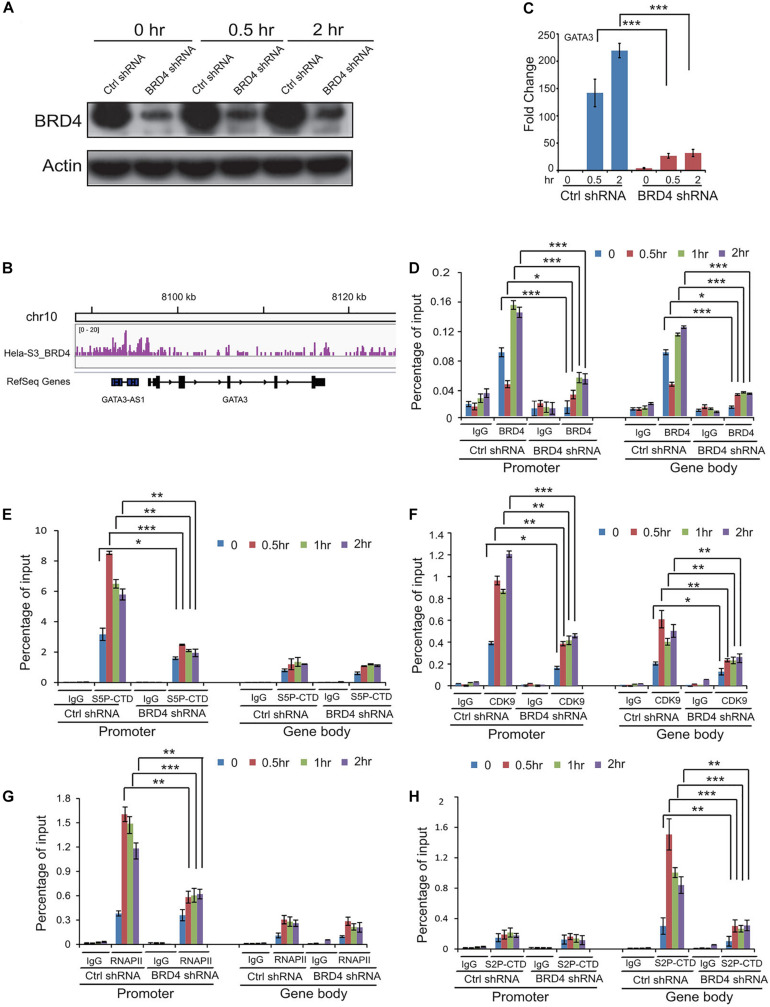
Dynamic recruitments of BRD4, P-TEFb, and RNAPII onto gene promoter and gene body of BRD4 positively regulated gene *GATA3* after UV stress. **(A)** *BRD4* knockdown efficiency in cells at 0, 0.5, and 2 h after UV treatment. **(B)** Representative genomic loci showing BRD4 binding at *GATA3* locus. **(C)** RT-PCR analysis of UV-upregulated gene *GATA3* expression after *BRD4* knockdown. **(D)** ChIP-qPCR analysis of isolated DNA associated with BRD4 at both promoter and gene body of *GATA3* in control and BRD4-depleted HeLa cells after UV treatment. **(E)** ChIP-qPCR analysis of isolated DNA associated with Ser5-CTD of RNAPII at both promoter and gene body of *GATA3* in control and BRD4-depleted HeLa cells after UV treatment. **(F)** ChIP-qPCR analysis of isolated DNA associated with CDK9 at both promoter and gene body of *GATA3* in control and BRD4-depleted HeLa cells after UV treatment. **(G)** ChIP-qPCR analysis of isolated DNA associated with RNAPII at both promoter and gene body of *GATA3* in control and BRD4-depleted HeLa cells after UV treatment. **(H)** ChIP-qPCR analysis of isolated DNA associated with Ser2-CTD of RNAPII at both promoter and gene body of *GATA3* in control and BRD4-depleted HeLa cells after UV treatment. The data in **(C)** to **(H)** are reported as mean values ± SD with the indicated significance by using Student’s *t* test (*p < 0.05, **p < 0.01, ***p < 0.001).

To study the dynamic associations of BRD4, P-TEFb, and RNAPII on BRD4-responsive genes after UV stress, chromatin was prepared from control and BRD4-depleted cells at 0, 0.5, 1, and 2 h after UV treatment (Figure 5A), and precipitated with anti-BRD4, anti-CDK9, and anti-RNA pol II antibodies. We examined the expression of *GATA3* that was upregulated after UV treatment and downregulated by *BRD4* knockdown (Figures 5B,C). ChIPed DNA was amplified for the *GATA3* promoter and gene body, showing an increase at 1 and 2 h in control cells after UV treatment (Figure 5D). These data are consistent with the UV-mediated dynamic association of BRD4 with chromatin. However, in BRD4-depleted cells, the recruitment of BRD4 to the *GATA3* promoter and gene body was significantly reduced after UV treatment.

Phosphorylation of RNAPII CTD has been implicated in several post-initiation regulatory steps (Kobor and Greenblatt, 2002). ChIP experiments with an antibody recognizing the phosphorylated form of Ser5 (S5P-CTD) revealed that Ser5-phosphorylated RNAPII peaks at the promoter and is present at the gene body of *GATA3* (Figure 5E). Interestingly, the Ser5 phosphorylation level at the promoter was increased to a higher extent than the total RNAPII levels upon UV stress induction, indicating that pre-bound RNAP II is not fully phosphorylated at Ser5 before activation (Figure 5G). In agreement with previous studies (Komarnitsky et al., 2000; Gomes et al., 2006), Ser2 phosphorylation of RNAPII CTD is not present at the promoters and accumulates in the body of *GATA3* (Figure 5H). UV stress induced even higher levels of CDK9 in the promoter and Ser2 phosphorylation in the gene body of *GATA3* compared with the control (Figures 5F,H). Furthermore, *BRD4* knockdown significantly reduced the UV-stress-induced recruitment of CDK9 and Ser2 phosphorylation, but not Ser5 phosphorylation (Figures 5E,F,H).

### Dynamic Recruitments of P-TEFb, S5P-CTD, and S2P-CTD of RNAPII to BRD4 Negatively Regulated Gene *WFIKKN1* After UV Treatment

**FIGURE 6:**
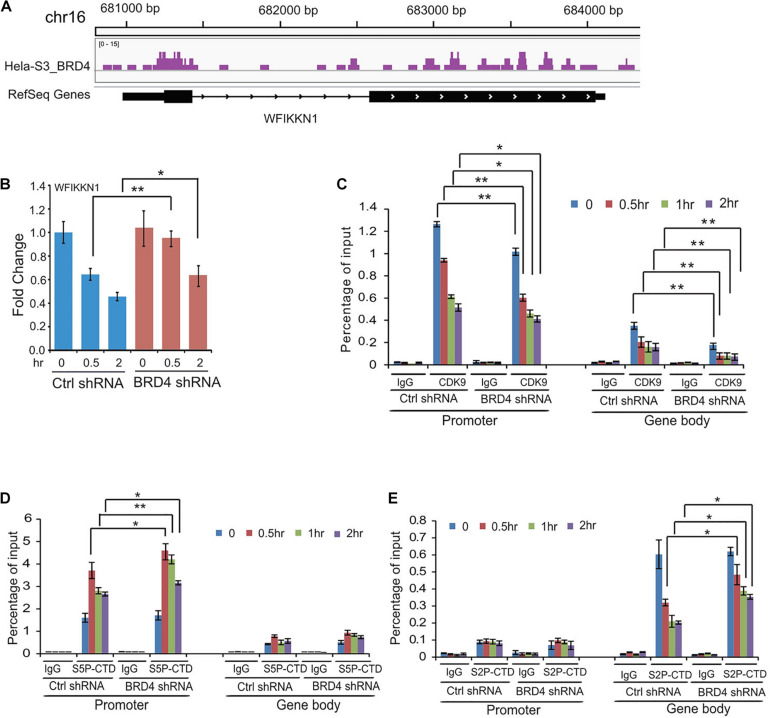
Dynamic recruitments of P-TEFb, Ser2-CTD, and Ser5-CTD of RNAPII onto gene promoter and gene body of BRD4 negatively regulated gene *WFIKKNI* after UV stress. **(A)** Representative genomic loci showing BRD4 binding at *WFIKKN1* locus. **(B)** RT-PCR analysis of UV-downregulated gene *WFIKKN1* expression after *BRD4* knockdown. **(C)** ChIP-qPCR analysis of isolated DNA associated with CDK9 at both promoter and gene body of *WFIKKNI* in control and BRD4-depleted HeLa cells after UV treatment (mean ± SD of *n* = 3). **(D)** ChIP-qPCR analysis of isolated DNA associated with Ser5-CTD of RNAPII at both promoter and gene body of *WFIKKNI* in control and BRD4-depleted HeLa cells after UV treatment (mean ± SD of *n* = 3). **(E)** ChIP-qPCR analysis of isolated DNA associated with Ser2-CTD of RNAPII at both promoter and gene body of *WFIKKNI* in control and BRD4-depleted HeLa cells after UV treatment (mean ± SD of *n* = 3). The data in **(B)** to **(E)** are reported as mean values ± SD with the indicated significance by using Student’s *t* test (*p < 0.05, **p < 0.01).

We found that multiple genes downregulated by UV stress were activated by *BRD4* knockdown, for example, *WFIKKN1* (Figures 6A,B). To test how P-TEFb, S5P-CTD, and S2P-CTD of RNAPII recruit these types of genes, we performed ChIP assays using antibodies against CDK9, S5P-CTD, and S2P-CTD. Following UV irradiation, we detected a decrease in CDK9 at the promoter of *WFIKKN1*, a gene negatively regulated by BRD4. The recruitment of CDK9 at the gene body of *WFIKKN1* was also reduced after *BRD4* knockdown, consistent with the BRD4-dependent recruitment of CDK9 (Figure 6C). S5P-CTD at the promoter of *WFIKKN1* was detected prior to UV irradiation. An increase in the S5P-CTD level at the promoter of *WFIKKN1* was observed after UV stress, and even higher after *BRD4* knockdown compared with control experiments (Figure 6D). In addition, the binding of S2P-CTD at the gene body of *WFIKKN1* was reduced at 0.5, 1, and 2 h after UV treatment (Figure 6E). Surprisingly, compared with control experiments, we observed a relative higher level in S2P-CTD at the gene body of *WFIKKN1* at 0.5, 1, and 2 h after UV stress by *BRD4* knockdown, in agreement with the gene expression after UV treatment in both the control and *BRD4* knockdown cells. These results suggest that the recruitment of S2P-CTD of RNAPII to this type of genes may be CDK9 independent and other kinases may replace CDK9 in regulating the S2P-CTD of RNAPII and the activity of this type of genes.

## Discussion

BRD4 binds acetylated histone H3 and H4 with the aid of both bromodomains throughout the cell cycle (Dey et al., 2003); therefore, BRD4 has been thought to play important roles in epigenetic memory. BRD4 interacts with P-TEFb in interphase, increases P-TEFb-dependent Ser2-CTD phosphorylation of RNAPII, and stimulates lineage-specific gene transcription (Zhang et al., 2012). Two separable P-TEFb complexes exist in the cell, one bound to BRD4 and the other bound to the inhibitory subunit composed of 7SK small nuclear RNA (7SK snRNA) and HEXIM1 (Jang et al., 2005; Yang et al., 2005). The binding of P-TEFb to either BRD4 or the inhibitory subunits occurs in a mutually exclusive manner and can be disrupted by external signals (such as HMBA or UV) (Yang et al., 2005; Ai et al., 2011). In this study, we investigated the detailed dynamic associations of BRD4 and P-TEFb with chromatin within 2 h after UV stress and found previously unknown and different chromatin associations for both BRD4 and P-TEFb in the nucleus.

The complete extraction of BRD4 from chromatin requires 0.3 M salt in normal untreated cells, whereas 0.2 M salt is sufficient to extract BRD4 from chromatin after an external signal, such as UV stress (Ai et al., 2011). In this study, we separated the nuclear soluble fraction and chromatin fraction by titration with different salt concentrations after UV treatment, and 0.15 M salt was chosen for further study (Supplementary Figure 1). We then used 0.15 M salt to investigate the dynamic associations of BRD4 and P-TEFb with chromatin in the nucleus of cells treated with UV at different time points. It has been reported that external signals (such as HMBA or UV) trigger the disassociation of P-TEFb from inactive 7SK snRNP and the release of BRD4 from acetylated histones (Chen et al., 2008; Ai et al., 2011). The signal-released BRD4 has in turn been reported to interact with the transcriptionally active form of P-TEFb (Ai et al., 2011). However, by analyzing the dynamics of BRD4 and P-TEFb with chromatin at different time points (within 2 h) after UV treatment, we interestingly obtained differentially dynamic associations of BRD4 and P-TEFb with chromatin under this condition. We found that the BRD4 released from chromatin within 30 min was then reloaded onto chromatin at 1 h after UV stress. However, P-TEFb interacts with chromatin more tightly immediately after UV stress because we observed that P-TEFb was gradually decreased in the soluble fraction within 2 h and was significantly increased in the chromatin fraction at 30 min after UV stress (Figure 1). These results suggest that the association of P-TEFb with chromatin is through a BRD4-independent mechanism at the early stage of UV stress, at least within 30 min after UV stress, even though BRD4 in general facilitates the recruitment of P-TEFb onto chromatin. It is obvious that the mere presence of BRD4 on chromosomes is insufficient to recruit P-TEFb. Although BRD4 is continuously present on chromosomes throughout the entire cell cycle, CDK9 is recruited only toward the end of mitosis. Another important question that arises from this study, which awaits further investigation, is which factor promotes the association of P-TEFb with chromatin within 30 min after UV stress because most chromatin-bound BRD4 is dissociated from chromatin and is still in the nuclear soluble fraction.

We surprisingly found that salt extracts more P-TEFb (CDK9 and Cyclin T1) into both the nuclear soluble and chromatin fractions after *BRD4* knockdown after UV stress, indicating that the interaction of P-TEFb with chromatin becomes much weaker after *BRD4* knockdown. The UV stress-mediated reloading of P-TEFb onto chromatin was much slower in BRD4-depleted cells compared with control cells (Figure 2). As the inhibitive partner of P-TEFb, UV stress results in more HEXIM1 protein in the nuclear soluble fraction with equal amounts in both control and BRD4-depleted cells. This result indicates that the slower recruitment of P-TEFb onto chromatin was not due to the HEXIM1 inhibitory effect. Our results indicated that *BRD4* knockdown has no effect on the UV-mediated dynamic associations of total RNAPII and Ser5 phosphorylation of RNAPII CTD onto the chromatin. However, *BRD4* knockdown significantly reduces the UV-induced Ser2 phosphorylation of RNAPII CTD, suggesting that BRD4 is required for the Ser2 phosphorylation of RNAPII CTD before and after UV stress.

BRD4 is released from chromosomes upon exposure to anti-mitotic drugs in a manner dependent on the activation of the JNK pathway (Nishiyama et al., 2012). The JNK pathway is one of three MAPK pathways (JNK, MEK, and p38). We identified that a JNK inhibitor but not a MEK or p38 inhibitor inhibits the interactions between BRD4 and CDK9/cyclin T1 at 2 h after UV treatment (Figure 3), suggesting that the JNK pathway is involved in the interactions between BRD4 and P-TEFb. These results also support the idea that JNK may act as a critical mediator of BRD4 release and help to protect cells against UV-induced disassociation of BRD4 and P-TEFb.

To activate gene expression, BRD4-mediated P-TEFb recruitment has been shown to target specific gene promoters (Mochizuki et al., 2008). Furthermore, BRD4 can recruit P-TEFb to a naked DNA template *in vitro* (Yang et al., 2005), indicating that factors other than acetylated nucleosome play a role. It has also been reported that BRD4 co-purifies with mediator components (Jiang et al., 1998; Jang et al., 2005). Based on these observations, it is likely that in addition to acetylated nucleosomes, adaptors such as mediators and/or sequence-specific transcription factors can be recruited by BRD4/P-TEFb complex to specific promoters for transcriptional activation (Lee et al., 2017). Indeed, qRT-PCR analysis indicated that UV-upregulated gene *GATA3* is positively affected by the BRD4 level (Figure 5), and ChIP assays confirmed that the recruitment of P-TEFb and phosphorylated Ser2-CTD of RNAPII to *GATA3* is BRD4-dependent. However, BRD4 has also been identified in a transcriptional silencing complex assembled by HPV E2 and is a corepressor that inhibits the expression of HPV-encoded E6 and E7 oncoproteins, which antagonize the activities of the p53 and pRB tumor suppressors, respectively (Wu et al., 2006). We found that a good number of the genes identified in the RNA-seq data as downregulated by UV stress were activated by *BRD4* knockdown, suggesting that BRD4 acts as a transcriptional repressor in regulating this group of genes by UV stress. Our finding indicates that BRD4 can be a component of either a transcriptional activation complex or a transcriptional silencing complex and regulate gene transcription in a context-dependent manner.

Although the binding of P-TEFb onto BRD4-negatively regulated gene *WFIKKN1* was significantly reduced after *BRD4* knockdown, the recruitment of phosphorylated RNAPII onto both the gene promoters (Ser5-CTD) and gene bodies (Ser2-CTD) was not influenced by *BRD4* knockdown (Figure 6). RNAPII CTD phosphorylation at Ser2 is independent of CDK9 but largely requires only CDK12 in the germline in *C. elegans* (Bowman et al., 2013). It will be interesting to explore which CDK is involved in Ser2-CTD phosphorylation of RNAPII on genes that are negatively regulated by BRD4 after DNA damage, especially UV stress.

## Materials and Methods

### Cell Culture

HeLa-S3 cells were cultured in Dulbecco’s Modified Eagle Medium (DMEM) supplemented with 10% fetal bovine serum (FBS) and 1% penicillin–streptomycin (Invitrogen). The cells were maintained in a 5% CO_2_ humidified incubator at 37°C.

### Antibodies

The following antibodies were used in this study: polyclonal anti-rabbit against BRD4 (ab75898), polyclonal anti-rabbit against RNA polymerase II (Santa Cruz Biotechnologies, sc-9001), polyclonal anti-rabbit against RNA polymerase II CTD repeat YSPTSPS (phospho S5) (ab5131), polyclonal anti-rabbit against RNA polymerase II CTD repeat YSPTSPS (phospho S2) (ab5095), polyclonal anti-rabbit against CDK9 (Santa Cruz Biotechnologies, sc-8338), polyclonal anti-rabbit against Cyclin T1 (Santa Cruz Biotechnologies, sc-10750), polyclonal anti-rabbit against HEXIM1 (ab25388), polyclonal anti-rabbit against Histone H3 (ab1791), and monoclonal anti-mouse against TFIIB (ab819).

### Lentiviral Production

A recombinant construct (pLKO.1 empty, pLKO.1-*BRD4*, pLKO.1-*MAPK1*, pLKO.1-*MAPK8*, pLKO.1-*MAPK14*), as well as two helper vectors, cytomegalovirus (CMV) R8.91 and VSV-G, were transiently transfected into 6 × 10^6^ cells/10 cm dish of 293T cells using Lipofectamine 2000. At 12 h after transfection, fresh medium was added. Viral supernatants were collected, filtered, and used to infect target cells after 48 h. The primers used for constructs are shown in Supplementary Table 1.

### Transfection

The collected viral supernatants were mixed with 8 μg/ml polybrene to infect target cells. Stable cell lines were selected with 1 μg/ml puromycin after the two infections for 48 h. The RNAi knockdown efficiencies were determined by Western blot and qRT-PCR.

### Isolation of Nuclear Soluble, Chromatin, and Matrix Insoluble Fraction

Cells were washed with PBS, scraped, and transferred to a Falcon tube. PBS was removed, and the cells were resuspended and incubated in 800 μl of buffer A (10 mM HEPES, 1.5 mM MgCl_2_, 10 mM KCl, 1 mM PMSF, 0.5 mM DTT, 10 mM NaF, 1 mMNa_3_VO_3_, and protease inhibitor) for 10 min on ice. The cells were pelleted by centrifuging at 1200 rpm for 5 min and resuspended in 200 μl of buffer A. 2.5 μl of 10% NP-40 was added. The pellet was resuspended by pipetting up and down and centrifuged for 5 min at 2000 rpm. The pelleted nuclei were resuspended in saponin buffer (100 mM KAC, 30 mM KCl, 10 mM Na_2_HPO_4_, 1 mM MgCl_2_, 1 mM disodium ATP, 1 mM DTT, 100 μg/ml saponin, and protease inhibitor) for swelling on ice for 5 min. The nuclear soluble fraction was collected by centrifuging at 2000 rpm for 5 min. The pellets were resuspended in buffer C (20 mM HEPES, 1.5 mM MgCl_2_, 420 mM NaCl, 25% glycerol, 1 mM PMSF, 0.5 mM DTT, 10 mM NaF, 1 mM Na_3_VO_3_, and protease inhibitor) supplemented with 0.05% NP-40. The sample was stirred for 30 min at cold room. The chromatin-bound proteins were collected by centrifuging at 13,000 rpm for 5 min.

### Flag co-IP (Immunoprecipitation)

Nuclear extract (NE) (600 μg) from cells overexpressing either Flag or Flag-*BRD4* was incubated with anti-Flag M2 dynabeads for 12 h at 4°C. The complex was washed with TBS buffer (50 mM Tris–HCl and 150 mM NaCl, pH 7.4), and the bound materials were eluted by FLAG peptides or in a sample buffer. The separated protein fractions were subjected to SDS-PAGE and transferred to a PVDF membrane (Invitrogen). Immunoblotting was performed with the indicated antibodies.

### RNA Sequencing (RNA-seq)

HeLa-S3 cells stable expressing Ctrl shRNA and *BRD4* shRNA were treated with 60 J/cm^2^ ultraviolet (UV) light. The cultured cells were collected at 0, 0.5, 1, and 2 h after UV treatment. Then, the cells were harvested and dissolved in Trizol for total RNA extraction and treated with DNase I (Ambion) to remove any potential contaminated DNA fraction. The following library generation and RNA-seq were conducted by WuXi AppTec and RiboBio. The reads were aligned to human hg19 genome transcriptome (Ensembl v73) using RSEM (Li and Dewey, 2011) and normalized for GC-content with EDASeq (Risso et al., 2011). Heatmap was generated by using Glbase (Hutchins et al., 2014) and gene ontology and pathway analysis were performed using clusterProfiler (Huang da et al., 2009).

### ChIP-seq

Raw fastq files were first subjected to Trim Galore^1^ to remove adaptors and low-quality reads. Trimmed reads were mapped to human hg19 genome using Bowtie2 (v2.2.5) (Langmead and Salzberg, 2012) with parameter “–very-sensitive” and then converted to bam using samtools (v1.2) (Li et al., 2009). Only uniquely mapping (map quality > 30), deduplicated reads were used for analysis. Peaks were identified with MACS2 (v2.1.0) (Zhang et al., 2008) using the following parameters “-f BAM -g hs –nomodel –extsize 146 -p 0.01.” Peak annotation was performed with ChIPseeker (Yu et al., 2015).

### qRT-PCR Assays

Total RNA was extracted from cells using the Trizol reagent (Invitrogen), and RNA was treated with DNase I (Ambion) for 30 min at 37°C. cDNA was synthesized using random primer and reverse transcriptase (Toyobo). cDNA amplification was monitored with SYBR premix Ex Taq (TAKARA) in a CFX96 real-time PCR system (BIO-RAD) according to the manufacturer’s instructions. The transcript levels were normalized to HPRT1. The primers used for qRT-PCR are shown in Supplementary Table 2.

### ChIP Followed by qPCR

ChIP was performed using the indicated antibodies. Briefly, cells (1 × 10^6^) were cross-linked with 1% paraformaldehyde for 10 min at 37°C and lysed in the buffer containing 50 mM Tris (pH 8.0), 10 mM EDTA, 1% SDS, and protease inhibitors. Chromatin was sheared by sonication to generate 200- to 1000-bp DNA fragments and used for immunoprecipitation steps. The indicated antibodies were incubated with Dynabeads A/G (Invitrogen) with rotation for 30 min at room temperature. The chromatin preparations were then incubated with the complex of antibody/magnetic beads overnight at 4°C. The immune complexes were washed twice each with low-salt buffer, high-salt buffer, LiCl buffer, and TE buffer. Antibody-bound chromatin was reverse-crosslinked. The ChIP DNA samples were purified and used for qPCR. The PCR primers used for ChIP are listed in Supplementary Table 3.

### Accession Codes

RNA-seq data described in this study have been deposited at the Gene Expression Omnibus (GEO) under accession GSE63385.

## Data Availability Statement

The dataset presented in this study can be found in an online repository. The name of the repository and accession number can be found below: Gene Expression Omnibus (GEO) – cGSE63385.

## Author Contributions

HY initiated the study and designed the experiments and conceived and supervised the entire study. YS conducted most of the experiments. GH and JJ performed the bioinformatics analysis. MY, XW, WL, AH, JC, and KO contributed to the work. HY, YS, and GH wrote the manuscript. All authors contributed to the article and approved the submitted version.

## Conflict of Interest

The authors declare that the research was conducted in the absence of any commercial or financial relationships that could be construed as a potential conflict of interest.

## Footnotes

1 http://www.bioinformatics.babraham.ac.uk/projects/trim_galore/

## Supplementary Material

Supplementary Figure 1 Association of BRD4 with chromatin at different salt concentrations under UV treatment.

Supplementary Table 1 Sequence of primers used for constructs of pLKO.1-*BRD4*, pLKO.1-*MAPK1*, pLKO.1-*MAPK8*, pLKO.1-*MAPK14*.

Supplementary Table 2 Sequence of primers used for qRT-PCR.

Supplementary Table 3 Sequence of primers used for ChIP-qPCR.
