# Supplementary figures and images for: DNA Damage Induces Dynamic Associations of BRD4/P-TEFb With Chromatin and Modulates Gene Transcription in a BRD4-Dependent and -Independent Manner
Source: Front Mol Biosci. 2020 Dec 4;7:618088. doi: 10.3389/fmolb.2020.618088 (PMC7746802; doi:10.3389/fmolb.2020.618088)

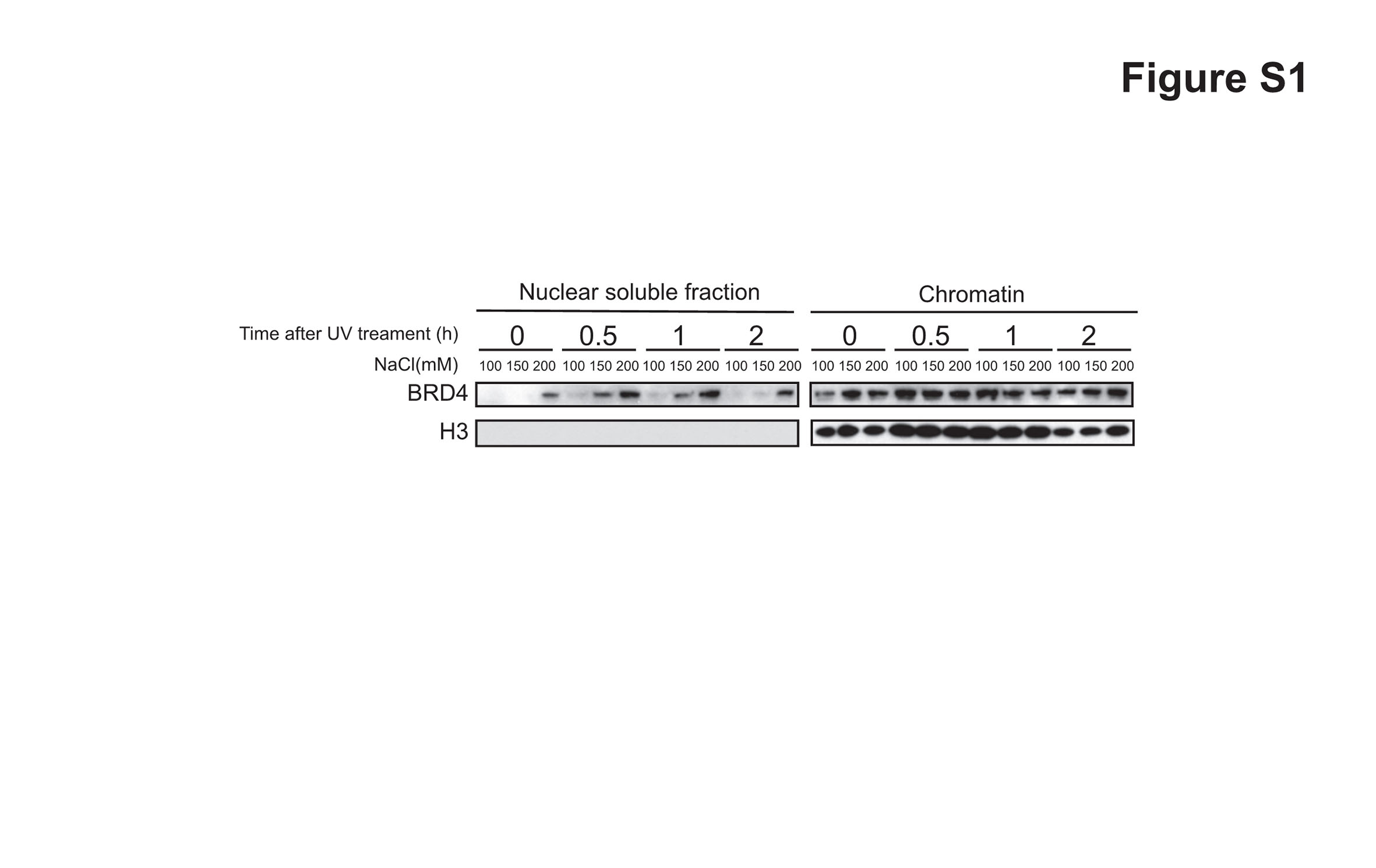

Supplement: Supplementary Figure 1 — Association of BRD4 with chromatin at different salt concentrations under UV treatment. [file Image_1.JPEG]
