# Supplementary material for: DNA Damage Induces Dynamic Associations of BRD4/P-TEFb With Chromatin and Modulates Gene Transcription in a BRD4-Dependent and -Independent Manner
Source: Front Mol Biosci. 2020 Dec 4;7:618088. doi: 10.3389/fmolb.2020.618088 (PMC7746802; doi:10.3389/fmolb.2020.618088)
Supplement: Supplementary Table 1 — Sequence of primers used for constructs of pLKO.1-BRD4, pLKO.1-MAPK1, pLKO.1-MAPK8, pLKO.1-MAPK14. [file Table_1.DOCX]

**Table S1. Sequence of primers used for constructs of pLKO.1-*BRD4*, pLKO.1-*MAPK1*, pLKO.1-*MAPK8*, pLKO.1-*MAPK14*.**

| **Gene name** | **Forward primer** | **Reverse primer** |
| --- | --- | --- |
| pLKO.1-*BRD*4 | CCGGGAACCTCCCTGATTACTATAACTCGAGTTATAGTAATCAGGGAGGTTCTTTTTG | AATTCAAAAAGAACCTCCCTGATTACTATAACTCGAGTTATAGTAATCAGGGAGGTTC |
| pLKO.1-*MAPK1* | CCGGCAAAGTTCGAGTAGCTATCAACTCGAGTTGATAGCTACTCGAACTTTGTTTTTG | AATTCAAAAACAAAGTTCGAGTAGCTATCAACTCGAGTTGATAGCTACTCGAACTTTG |
| pLKO.1-*MAPK8* | CCGGCAGTAAGGACTTACGTTGAAACTCGAGTTTCAACGTAAGTCCTTACTGTTTTTG | AATTCAAAAACAGTAAGGACTTACGTTGAAACTCGAGTTTCAACGTAAGTCCTTACTG |
| pLKO.1-*MAPK14* | CCGGGCCGTATAGGATGTCAGACAACTCGAGTTGTCTGACATCCTATACGGCTTTTTG | AATTCAAAAAGCCGTATAGGATGTCAGACAACTCGAGTTGTCTGACATCCTATACGGC |
