# Supplementary material for: DNA Damage Induces Dynamic Associations of BRD4/P-TEFb With Chromatin and Modulates Gene Transcription in a BRD4-Dependent and -Independent Manner
Source: Front Mol Biosci. 2020 Dec 4;7:618088. doi: 10.3389/fmolb.2020.618088 (PMC7746802; doi:10.3389/fmolb.2020.618088)
Supplement: Supplementary Table 2 — Sequence of primers used for qRT-PCR. [file Table_2.DOCX]

**Table S2. Sequence of primers used for qRT-PCR.**

| **Gene name** | **Forward primer** | **Reverse primer** |
| --- | --- | --- |
| *WFIKKN1* | CCACTTCCCCACACCTTGTC | GCCTCGTAGGTCTCAAAGCC |
| *GATA3* | GGCTTCGGATGCAAGTCCAG | AGCCTTCGCTTGGGCTTAAT |
